# Differentiation of Human Induced Pluripotent Stem Cells (iPSCs)–derived Mesenchymal Progenitors into Chondrocytes

**DOI:** 10.21769/BioProtoc.4874

**Published:** 2023-11-05

**Authors:** Nazir M. Khan, Martha Elena Diaz-Hernandez, Hicham Drissi

**Affiliations:** 1 Department of Orthopaedics, Emory University, Atlanta, GA, USA; 2 Atlanta VA Medical Center, Decatur, GA, USA

**Keywords:** Induced pluripotent stem cells (iPSCs), Mesenchymal stromal/stem cells (MSCs), Chondrogenic differentiation, High density culture, Chondrocytes

## Abstract

Induced pluripotent stem cells (iPSCs) generated from human sources are valuable tools for studying skeletal development and diseases, as well as for potential use in regenerative medicine for skeletal tissues such as articular cartilage. To successfully differentiate human iPSCs into functional chondrocytes, it is essential to establish efficient and reproducible strategies that closely mimic the physiological chondrogenic differentiation process. Here, we describe a simple and efficient protocol for differentiation of human iPSCs into chondrocytes via generation of an intermediate population of mesenchymal progenitors. These methodologies include step-by-step procedures for mesenchymal derivation, induction of chondrogenic differentiation, and evaluation of the chondrogenic marker gene expression. In this protocol, we describe the detailed procedure for successful derivation of mesenchymal progenitor population from human iPSCs, which are then differentiated into chondrocytes using high-density culture conditions by stimulating with bone morphogenetic protein-2 (BMP-2) or transforming growth factor beta-3 (TGFβ-3). The differentiated iPSCs exhibit temporal expression of cartilage genes and accumulation of a cartilaginous extracellular matrix in vitro, indicating successful chondrogenic differentiation. These detailed methodologies help effective differentiation of human iPSCs into the chondrogenic lineage to obtain functional chondrocytes, which hold great promise for modeling skeletal development and disease, as well as for potential use in regenerative medicine for cell-based therapy for cartilage regeneration.

Key features

• Differentiation of human iPSCs into chondrocytes using 3D culture methods.

• Uses mesenchymal progenitors as an intermediate for differentiation into chondrocytes.

## Background

Human induced pluripotent stem cells (iPSCs) hold great potential for regenerative medicine, as they can be tailored to patient-specific cells for replacing musculoskeletal tissues with limited inherent repair capacity, such as articular cartilage. However, the pluripotent nature of human iPSCs poses a significant challenge in directing their differentiation towards specialized cell types, such as chondrocytes. Various in vitro strategies have been reported for inducing chondrogenic differentiation of human pluripotent stem cells (Guzzo and Drissi, 2015; Wu et al., 2021; Dicks et al., 2023; Khan et al., 2023; Lamandé et al., 2023; Zujur et al., 2023). Some of these approaches involve initial pre-differentiation steps within embryoid bodies, followed by dispersal and high-density culture to promote cell–cell interactions that mimic pre-cartilage condensation during skeletal development (Guzzo and Drissi, 2015; Dicks et al., 2023). Other studies have investigated the efficacy of stage-specific administration of developmentally relevant growth factors, such as bone morphogenetic proteins (BMPs) and transforming growth factors (TGFs), in controlling the induction of human pluripotent stem cells into the chondrogenic lineage and subsequent chondrocyte differentiation (Suchorska et al., 2017; Mahboudi et al., 2018).

We have previously developed a method to induce chondrogenic differentiation from human iPSCs using mesenchymal-like progenitor cells as the intermediate population (Guzzo et al., 2013; Guzzo and Drissi, 2015). These progenitor-like cells exhibit molecular and functional properties similar to adult mesenchymal stem cells (MSCs) as a means to limit the developmental potency of iPSCs (Guzzo et al., 2013; Guzzo and Drissi, 2015; Khan et al., 2023). A readily expandable source of iPSCs-derived multipotent progenitors (iMSCs) with high chondrogenicity could potentially provide a vast supply of cells for regenerative medicine applications, as they can be used as an alternate source of adult MSCs, such as bone-marrow-derived MSCs and adipose-derived stem cells. These MSCs derived from iPSCs (referred here as *iMSCs*) have the potential to overcome clinical barriers for therapeutic applications of adult MSCs, as these iMSCs represent infinitive cell sources (ex vivo and in vivo) as well as isogenic MSCs providing renewable cell sources for MSCs.

**Figure 1.**
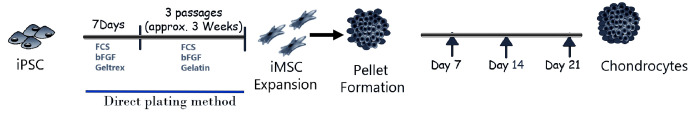
Graphical abstract showing different stages of induced pluripotent stem cells (iPSC) differentiation into mesenchymal progenitors (iMSCs) and chondrogenic differentiation using direct 3D pellet culture method

Here, we provide detailed step-by-step protocols for chondrogenic differentiation of human iPSCs from a naive, pluripotent state and via the derivation of a scalable, mesenchymal-like progenitor intermediate as we have described previously (Khan et al., 2023). We first describe the derivation of iMSCs from human iPSCs using direct plating and embryoid bodies formation. We then describe the differentiation of iMSCs into chondrocytes using 3D pellet culture and micromass culture method. We also describe the histological methods and gene expression analyses used to evaluate chondrogenic differentiation. The schematic representation of the step-by-step method used for differentiation of iPSCs to chondrocytes is shown in Figure 1.

## Materials and reagents

**Biological materials**

1. Human dermal fibroblast-derived iPS cells (YK27 #10.1)
2. Human chondrocytes-derived iPSCs (AC-iPSC) (generated in lab) (Khan et al., 2023)

**Reagents**

**Cell culture reagents**

1. mTeSR^TM^ Plus medium (Stem Cell Technologies, catalog number: 100-1130)
2. Geltrex^TM^ (Gibco, catalog number: A15696-01)
3. Anti-Adherence Rinsing Solution (Stem Cell Technologies, catalog number: 07010)
4. DPBS without calcium and magnesium (1×) (Gibco, catalog number: 14190-144)
5. ReLeSR (Stem Cell Technologies, catalog number: 05872)
6. Rho-associated kinase (Rock) inhibitor Y-27632 (Stem Cell Technologies, catalog number: 72302)
7. Dimethyl sulfoxide (DMSO) (Sigma-Aldrich, catalog number: D5879)
8. DMEM-high glucose (with 4.5 g/L D-glucose, with L-Glutamine, without sodium pyruvate) (Gibco, catalog number: 11965-092)
9. Defined fetal bovine serum (FBS) (Hyclone, catalog number: SH30070.03)
10. GlutaMAX (100×) (Gibco, catalog number: 35050061)
11. Non-essential amino acids solution (NEAA), 100× (Gibco, catalog number: 11140050)
12. HEPES buffer solution (1 M) (Gibco, catalog number:15630-080)
13. Penicillin-Streptomycin (100×) (Gibco, catalog number: 15140122)
14. Ham’s F12 nutrient mix (Gibco, catalog number: 11765054)
15. 0.05% Trypsin-EDTA (1×) (Gibco, catalog number: 25300062)
16. Sodium pyruvate (100 mM) (Gibco, catalog number: 11360070)
17. L-Ascorbic acid (Sigma, catalog number: A92902)
18. Trypan Blue solution 0.4% (Gibco, catalog number: 15250061)
19. L-Proline (Sigma, catalog number: P5607)
20. Dexamethasone (Sigma, catalog number: D2915)
21. ITS^TM^ Premix solution (Corning, catalog number: 354350)
22. Recombinant human FGF-basic (154 a.a.) (bFGF) (Peprotech, catalog number: 100-18B)
23. Recombinant human Bone morphogenetic protein-2 (BMP-2) (Peprotech, catalog number: 120-02C)
24. Recombinant human transforming growth factor beta-3 (TGF-β3) (Peprotech, catalog number: 100-36E)
25. 7.5% bovine serum albumin (BSA) solution (Sigma, catalog number: A8412)
26. Gelatin (0.1% in water) (Stem Cell Technologies, catalog number: 07903)
27. 70% ethanol (vol/vol) (made in laboratory)
28. Formaldehyde solution (vol/vol) (Sigma, catalog number: 252549)
29. 10% neutral buffered formalin (Azer Scientific, catalog number: PFNBF-0.6G)
30. Paraffin (Leica Biosystems, catalog number: 39601095)

**Molecular biology reagents**

1. TRIzol^®^ (Ambion, catalog number: 15596018)
2. Nuclease-free water (Ambion, catalog number: AM9906)
3. Isopropanol, molecular biology grade (Fisher Scientific, catalog number: BP2618500)
4. Ethanol, molecular biology grade (Fisher Scientific, catalog number: BP28184)
5. Chloroform (Sigma, catalog number: C2432)
6. High-Capacity cDNA Reverse Transcription kit (Thermo Fisher Scientific, catalog number: 4368814)
7. PowerUp SYBR Green Master Mix (Thermo Fisher Scientific, catalog number: A25742)
8. Custom primer (Integrated DNA Technologies) (Sequences of primer were given in table)
9. DNase I, RNase-free (Thermo Fisher Scientific, catalog number: EN0525)

**Histology reagents**

1. Xylene, histology grade (VWR, catalog number: 89370-088)
2. Acetic acid solution 3% aqueous (Poly Scientific R&D Corp, catalog number: s101-16 oz)
3. Alcian Blue solution (Poly Scientific R&D Corp, catalog number: s111A-16 oz)
4. Nuclear Fast Red Kernechtrot solution (Poly Scientific R&D Corp, catalog number: s248-32 oz)
5. Safranin O (Electronic Microscopy Sciences, catalog number: 20800)
6. CytoSeal mounting medium (Richard-Allen Scientific, catalog number: 8312-4)

**Antibodies**

Antibodies are used for quantifying MSC surface antigens:

1. APC Mouse Anti-Human CD34 (BD, catalog number: 560940)
2. V450 Mouse Anti-Human CD45 (BD, catalog number: 560368)
3. PE Mouse Anti-Human CD73 (BD, catalog number: 561014)
4. FITC Mouse Anti-Human CD90 (BD, catalog number: 555595)
5. PerCP-Cy5.5 Mouse Anti-Human CD105 (BD, catalog number: 560819)

**Solutions**

1. L-Proline solution (see Recipes)
2. Dexamethasone solution (see Recipes)
3. Ascorbic acid solution (see Recipes)
4. 0.1% Bovine serum albumin (BSA) solution (see Recipes)
5. Fibroblast growth factor-basic (bFGF) solution (see Recipes)
6. Bone morphogenetic protein-2 (BMP-2) stock solution (see Recipes)
7. Transforming growth factor beta-3 (TGF-β3) stock solution (see Recipes)
8. MSC growth media solution (see Recipes)
9. MSC freezing media solution (see Recipes)
10. Chondrogenic differentiation media (see Recipes)
11. Rho-associated kinase (ROCK) inhibitor Y-27632 (see Recipes)

**Recipes**

1. **L-Proline solution**
  4 mg/mL solution (100× stock) made in water
  Filter sterilize by passing it through a 0.22 μm filter
  Aliquot and store at ≤ -20 °C
2. **Dexamethasone solution**
  100 mM dexamethasone solution (100,000× stock) in sterile water
  Filter sterilize by passing it through a 0.22 μm filter
  Aliquot and store at ≤ -20 °C
3. **Ascorbic acid solution**
  16.67 mg/mL solution (333× stock) in sterile water
  Filter sterilize by passing it through a 0.22 μm filter
  Aliquot and store at ≤ -20 °C
4. **0.1% Bovine serum albumin (BSA) solution**
  0.1% BSA solution in sterile DPBS
  Aliquot and store at 4°C
5. **Fibroblast growth factor-basic (bFGF) solution**
  10 μg/mL of human recombinant bFGF in sterile DPBS containing 0.1% BSA.
  Aliquot and store at ≤ -80 °C
6. **Bone morphogenetic protein-2 (BMP-2) stock solution**
  100 μg/mL of human recombinant BMP-2 in sterile PBS containing 0.1% BSA. Aliquot and store at -80 °C.
7. **Transforming growth factor beta-3 (TGF-β3) stock solution**
  20 μg/mL of human recombinant TGF-β3 in sterile PBS containing 0.1% BSA fraction V. Aliquot and store at -80 °C.
8. **MSC growth media**
  DMEM-high glucose
  10% defined FBS
  1× Pen-Strep
  1× NEAA
  5 ng/mL b-FGF
  Store at 4 °C. Use within a month of preparation.
9. **MSC freezing media (Cryopreservation media)**
  80% defined FBS
  10% MSC growth media
  10% DMSO
10. **Chondrogenic differentiation Media**
  DMEM-high Glucose
  25 mM HEPES
  100 nM Dexamethasone
  50 μg/mL ascorbic acid
  1% ITS Premix
  40 μg/mL L-Proline
  1 mM sodium pyruvate
  1× NEAA
  1× GlutaMAX^TM^
  1× Pen-Strep
  100 ng/mL BMP-2 (added just prior to use)
  10 ng/mL TGFβ-3 (added just prior to use)
11. **Rho-associated kinase (ROCK) inhibitor Y-27632 solution**
  10 mM (1,000× stock) Y-27632 in sterile water
  Aliquot and store at -20 °C

**Laboratory supplies**

1. 15 and 50 mL conical tubes (Thermo Scientific, catalog numbers: 339650, 339652)
2. 1.5 and 0.5 mL centrifuge tubes (USA Scientific, catalog numbers: 1615-5510, 1605-0000)
3. 48-multiwell, 24-well, and 6-well tissue culture plates (USA Scientific, catalog numbers: CC7682-7548, CC7682-7524, CC7682-7506)
4. 100 and 60 mm tissue culture dishes (Corning, catalog numbers: 430293, 430196).
5. 1, 5, 10, and 25 mL disposable plastic serological pipettes (Corning, catalog numbers: 4012, 4051, 4101, 4251)
6. Low-retention graduated barrier pipette tips (10, 20, 200, and 1,000 μL) (MIDSCI, catalog numbers: PR-10RK-FL, PR-20RK-FL, PR-200RK-FL, PR-1000RK-FL)
7. 0.22 and 0.45 μm disposable sterile filters (Millipore, catalog numbers: SLGV033RB, SLHV033RS)
8. 1.5 mL cryogenic vials (Nalgene, catalog number: 5000-1020) and cryovial storage racks
9. 70 μm cell strainer (Falcon, catalog number: 352350 or equivalent)
10. 1 mL syringe with 26 1/2-gauge needle 1 (BD Biosciences, catalog number: 309625)

## Equipment

1. Pipette aid (Eppendorf, catalog number: 4430000018)
2. Inverted phase-contrast microscope (4×, 10×, 40× objectives) (Nikon, model: TMS 215135)
3. Tissue culture centrifuge with multiple rotors (Eppendorf, model: 5804R)
4. Humidified CO_2_ incubators (Forma Scientific, model: 3110)
5. Airclean 600 PCR workstation
6. Picking microscope (Leica S9D)
7. Water bath (LAB-LINE, AQUABATH; model: 16070)
8. Vortexer (BIO-RAD, model: BR-2000)
-80 °C freezers (Panasonic, model: MDF-U53VA-PA)
10. Liquid nitrogen tank (Locator JR Plus Cryo Biological Storage System)
11. Hemocytometer (Brigh-Line, model: 1492)
12. Biosafety cabinet (StreilGARD III Advance, The Baker Company, model: Model SG603)
13. Flow cytometer (BD Biosciences, model: BD-Accuri-C6)
-20 °C freezer (Frigidiare Commercial)
15. NanoDrop (Thermo Scientific, model: 2000/2000C)
16. Real time quantitative reverse transcription PCR (RT-PCR) (Applied Biosystem, model: 7500)
17. Heating block (VWR Scientific, model: 949030)
18. Mr. Frosty^TM^ Freezing Container (Thermo Scientific, catalog number: 5100-0036)

## Software and datasets

1. Flow Jo (Version 10)
2. ImageJ (Version 1.5.1)
3. 7500 software (Version 2.1.0)

## Procedure

- **Thawing and recovering human iPSCs**
  1. Pre-warm an appropriate volume of mTeSR^TM^ Plus medium to room temperature in a 50 mL Falcon tube (refer to Note 1).
  2. Prepare the Geltrex-coated 6-well plates at least 1 h before iPSC thawing.
  3. Retrieve the cryogenic vial containing frozen iPSCs from the liquid nitrogen promptly and place it in a 37 °C water bath.
  4. Hold the cap of the cryogenic vial and gently swirl it to thaw the cells, ensuring that the cap remains above the water level.
  5. Once most cells are thawed and only a few ice crystals remain, remove the cryogenic vial from the water bath, spray it with 70% ethanol, and transfer it to a laminar hood.
  6. Carefully open the cap of the vial and transfer the cell suspension into a 15 mL Falcon tube containing the 10 mL pre-warmed Ham’s F12 nutrient mix.
  7. Rinse the cryogenic vial with 1 mL of F12 medium and add the solution to the same Falcon tube, gently mixing the cells by pipetting.
  8. Centrifuge the Falcon tube at 300× *g* for 5 min at room temperature and discard the supernatant.
  9. Gently resuspend the iPSC pellets in 2 mL of mTeSR^TM^ Plus medium by gently pipetting up and down 3–4 times using 1 mL pipette tips, being cautious not to break the iPSC colonies into single-cell suspension.
  10. Add 2 μL of ROCK inhibitor (1,000× stock solution) to the cell suspension to enhance iPSC survival (refer to Note 3).
  11. Prepare a 6-well plate by removing the excess Geltrex from the pre-coated 6-well plate and then wash the wells with 2 mL of 1× DPBS. Aspirate the PBS and slowly transfer the iPSC suspension into the plate. Distribute one cryogenic vial of iPSCs into 1–2 wells of the 6-well plate.
  12. Gently tilt the culture plate back and forth and move it left and right several times to disperse cells evenly across the entire well (refer to Note 4).
  13. Incubate the plate in a 37 °C, 5% CO_2_ incubator and allow the cells to culture overnight.
  14. The following day, replace the medium with fresh mTeSR^TM^ Plus medium, excluding the Rock inhibitor Y-27632.
  15. Subsequently, change media every day, replacing with fresh 2 mL of mTeSR^TM^ Plus medium per well in the 6-well plate. Allow the iPSCs to grow for 4–5 days. Upon reaching 70%–80% confluence, the cells can be passaged (refer to Note 5).
- **Passage iPSCs using ReLeSR method**
  Typically, we pass iPSCs every 4–5 days, when they reach a confluence of 70%–80% and can be passaged as cell aggregates. For the passaging of iPSCs as cell aggregates, ReLeSR is routinely employed in our protocols (refer to Note 6). mTeSR^TM^ is an enzyme-free reagent that selectively detaches undifferentiated cells and generates optimal-size aggregates.
  1. Before initiating the protocol, ensure that new 6-well plates are coated with Geltrex at least 1 h in advance. Additionally, pre-warm the required volume of mTeSR^TM^ Plus medium to room temperature.
  2. Carefully aspirate the spent medium from the wells containing cells and perform a single wash with 2 mL of DPBS without Ca^2+^ and Mg^2+^. No selective removal of differentiated regions of iPSCs is necessary.
  3. Add 1 mL of ReLeSR into each well of the 6-well plate. Swirl the plate gently to ensure uniform distribution of ReLeSR over the entire cell surface. Within one minute, aspirate the ReLeSR solution from the wells.
  4. Add 1 mL of mTeSR^TM^ Plus medium to each well of the 6-well plate and monitor cell dissociation under an inverted microscope.
  5. Once iPSCs start to exhibit separation and rounding up at the periphery, they are ready to be removed from the wells using a cell lifter.
  6. Gently pipette the cell mixtures up and down 5–6 times to disperse large colonies into small clumps. Collect all resulting cell aggregates into a 15 mL tube (refer to Note 8).
  7. Centrifuge the 15 mL tube at 300× *g* for 5 min at room temperature and discard the supernatant. Add 2 mL of mTeSR^TM^ Plus medium to the cell aggregate in 15 mL tube.
  8. Prepare the 6-well plate by removing any remaining Geltrex solution from the pre-coated plate. Wash the plate once with 2 mL of 1× PBS to remove any remaining Geltrex.
  9. Transfer the 0.5 mL of mTeSR^TM^ Plus medium containing cell aggregate to each well of the 6-well plate and uniformly distribute the cell aggregate onto the plate surface. Add 1.5 mL of mTeSR^TM^ Plus medium to each well to make a total volume of 2 mL. Generally, after 80% confluence of iPSC colonies, we pass the cells into 1:4 split ratio.
  10. Supplement the culture system with 2 μL of Rock inhibitor Y-27632 (1,000×, working concentration 10 μM) to enhance the survival of iPSCs.
  11. Gently tilt and move the plate in quick back and forth and side to side motions to evenly distribute the cell aggregates across the plate surface. Subsequently, return the plate to the 37 °C incubator (refer to Note 10).
  12. Daily, refresh the mTeSR^TM^ Plus medium and observe the cultures under suitable conditions to monitor growth until the subsequent passaging or banking (refer to Note 11).
- **Derivation of mesenchymal progenitors (MSCs) from iPSCs using direct plating method**
  We have devised a streamlined and effective protocol for the feeder-free differentiation of iPSCs into MSCs in a controlled culture system.
  1. Before initiating the differentiation process, coat each well of a 6-well plate with 1 mL of 0.1% gelatin solution and allow it to incubate at room temperature for 1 h. Subsequently, wash the coated plates once with 1× PBS.
  2. Remove the mTeSR^TM^ Plus medium from the routine iPSC cultures in 6-well plates and wash the cells with sterile PBS. Apply 1 mL of ReLeSR to each well and gently swirl the plate to ensure even coverage of the cell surface. Within a minute, aspirate the ReLeSR solution from the wells.
  3. Add 1 mL of mTeSR^TM^ Plus medium to the wells and, using a cell lifter, remove the colonies from the plate. Gently pipette the cell mixtures up and down 3–4 times to break down large colonies into smaller clumps. Collect all resulting cell aggregates into a 15 mL tube.
  4. Centrifuge the 15 mL tube at 300× *g* for 5 min at room temperature and discard the supernatant. Wash the cell aggregates with 1× PBS and then add 1 mL of mesenchymal stem cell induction and growth medium (referred to as *MSC growth medium*) to each tube.
  5. Plate the iPSCs onto gelatin-coated 6-well plates. Employ a cell culture split ratio of 1:1 or 1:2, where cells harvested from one well are distributed onto one or two new wells. This step marks the initial passage, referred to as iPS-MSCs or iMSCs passage 0 (p0).
  6. Change the medium every 2–3 days. After 7–10 days, the cultures will exhibit a mixture of flattened cuboidal and elongated spindle-shaped cells, indicating successful differentiation (refer to Note 12). Once a confluent monolayer is attained, proceed to the next passage.
  7. Apply 0.5 mL of 0.25% Trypsin-EDTA solution to each well of a 6-well dish and incubate at 37 °C for 3–5 min. Use repeated pipetting with a 1 mL pipettor to singularize the cells. Transfer the cell suspension to a 15 mL conical tube containing MSC growth medium. Centrifuge the cells at 300× *g* for 5 min and discard the supernatant.
  8. Resuspend the cell pellet in MSC growth medium and perform cell counting. Prepare a cell suspension at a concentration of 0.25 × 10^6^ cells/mL. Plate 1 mL of the cell suspension into each well of a 6-well culture plate, which has been precoated with gelatin solution. This will mark the iPS cell-MSCs (iMSCs) passage 1.
  9. Maintain the cells in a humidified atmosphere with 5% CO_2_ at 37 °C, with medium changes every 2–3 days. Within two weeks, the cells will adopt a fibroblastic, spindle-like morphology indicative of mesenchymal progenitor cells (Figure 2) (refer to Note 13). When cultures reach 90% confluence, split the cells using 0.25% Trypsin-EDTA solution and seed them at a density of 1 × 10^4^ cells/cm^2^ onto gelatin-coated plates.
    **Figure 2.**
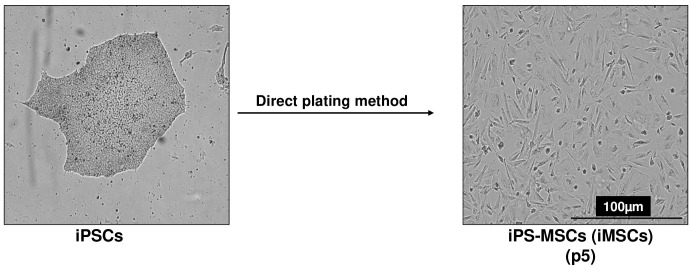
Differentiation of human induced pluripotent stem cells (iPSCs) into mesenchymal progenitors (iMSCs) using direct plating methods. Scale bar, 100 μm.
  10. Assay the expression of stem cell genes (e.g., Oct4, Nanog, alkaline phosphatase, Klf4) and gene markers associated with the mesenchymal lineage (e.g., Twist1, Col1a1) by quantitative RT-PCR, as described below in step G11. Notably, stem cell genes will be suppressed in the mesenchymal-like population, while mesenchymal markers will be significantly induced.
  11. For routine expansion, seed the cells at a density of 1 × 10^4^ cells/cm^2^ and maintain them in MSC growth medium (see Note 14). Keep track of the passage numbers. Establish a cell bank by freezing batches at each passage. At harvest, resuspend singularized cells in 1× cryopreservation medium and aliquot 1 × 10^6^ cells per vial. Freeze the vials at -80 °C and subsequently transfer them to liquid nitrogen for long-term storage (see Note 15).
- **Cryopreserving iPSC-MSCs (iMSCs)**
  Following serial trypsinization, the induced pluripotent stem cell–derived mesenchymal stem cells (iPSC-MSCs or iMSCs) exhibit an enhanced maturation state, rendering them amenable to cryopreservation after passages 3–5.
  1. Prepare fresh 2× MSC freezing medium and keep it on ice until use.
  2. Obtain a single cell suspension of MSCs using the Trypsin-EDTA method, following the procedures detailed in steps C7–C9.
  3. Gently dislodge the cell pellets through finger tapping and resuspend the cells in MSC growth medium to achieve a single-cell suspension.
  4. Using a hemocytometer, perform cell counting and adjust the cell density to 2 × 10^6^ cells/mL.
  5. Add an equal volume of cold 2× MSC freezing medium to the MSC cell suspension, ensuring thorough mixing.
  6. Aliquot 1 mL of the cell mixture into each cryogenic vial. Each cryogenic vial should contain 1 × 10^6^ cells (refer to Note 17).
  7. Swiftly place the cryogenic vials into the freezing container and maintain them at -80 °C overnight.
  8. The following day, transfer the cryogenic vials containing cells to a -80 °C freezer or a liquid nitrogen tank for sustained long-term storage.
- **MSC surface antigens analysis by flow cytometry**
  The iPSC-derived mesenchymal stem cells (iPSC-MSCs or iMSCs) exhibit comparable surface antigen expression to human bone marrow–derived MSCs (BM-MSCs). For the analysis of surface antigens, antibodies targeting human CD34, CD45, CD73, CD90, and CD105 can be employed (refer to Antibodies section). Human bone marrow–derived MSCs serve as positive control cells, while the negative markers include CD34- and CD45-, and the positive markers comprise CD73^+^, CD90^+^, and CD105^+^.
**Protocol for flow cytometric analysis of surface antigens**
  1. Prepare the MSC single-cell suspension as described in steps C7–C9.
  2. Gently break the cell pellets through finger tapping and resuspend the cells using cold 1% FBS in DPBS without Ca^2+^ and Mg^2+^.
  3. Count the total number of cells using a hemocytometer and adjust the cell density to 2 × 10^6^ cells/mL.
  4. Transfer 2 × 10^5^ cells (100 μL cell suspension) into 1.5 mL centrifuge tubes (refer to Note 18).
  5. Add the appropriate volume of MSC antibodies (refer to Antibodies section) to each tube and gently mix them by pipetting.
  6. Incubate the cell samples for 30 min at room temperature in the dark.
  7. After the incubation, wash the cells once with 1% FBS in DPBS without Ca^2+^ and Mg^2+^. Finally, resuspend the cell samples in 250 μL of 4% formaldehyde-DPBS without Ca^2+^ and Mg^2+^.
  8. Analyze the cell samples using a flow cytometer (refer to Note 20) to evaluate surface antigen expression as described in our previous paper (Khan et al., 2023).
- **Differentiation of iPSC-MSCs (iMSCs) into chondrocytes**
  The iPSC-MSCs (iMSCs) demonstrate a versatile capacity for multipotent differentiation encompassing osteogenesis, chondrogenesis, and adipogenesis. In this study, we explored two distinct in vitro methods for differentiating iMSCs into chondrocytes, as described below.
**Micromass method for differentiation of iMSCs into chondrocytes**
  1. Expand human iMSCs in a 10 cm culture dish in MSC growth medium at 37 °C and 5% CO_2_ until they reach 75%–80% confluence.
  2. Aspirate culture medium and wash the cells once with 10 mL of PBS. Subsequently, apply 1 mL of 0.25% Trypsin-EDTA solution to the culture dish (refer to Note 21). Return the dish to the 37 °C incubator and incubate for approximately 3–5 min. Upon detachment of the cells, pipette gently and repeatedly using a 1 mL pipettor to disperse the iMSCs into single cells.
  3. Transfer the cell suspension to a 15 mL sterile conical polypropylene tube containing MSC growth medium. Centrifuge the cells at 300× *g* for 5 min and discard the supernatant.
  4. Resuspend the cell pellet in 5 mL of MSC growth medium and perform gentle pipetting using a 1 mL pipettor to make single-cell suspension. If required, pass the cell suspension through a 40 μm nylon cell strainer.
  5. Cell counting is done using the Trypan Blue exclusion method with a hemocytometer and Trypan Blue solution or by employing an automated cell counter. Cells are subsequently diluted in MSC growth medium to achieve a final concentration of 25 × 10^6^ cells/mL.
  6. For seeding, apply 10 μL drops of the diluted cell suspension onto 6-well culture plates (see Note 22). Add up to three high-density cell spots per well of a 6-well dish, ensuring adequate spacing between the drops (Figure 3A) These micromasses represent structural condensation of iMSCs during chondrogenic differentiation. Figure 3A shows the formation of three micromasses, which are equally spaced in the 6-well plate (see Note 23).
    **Figure 3.**
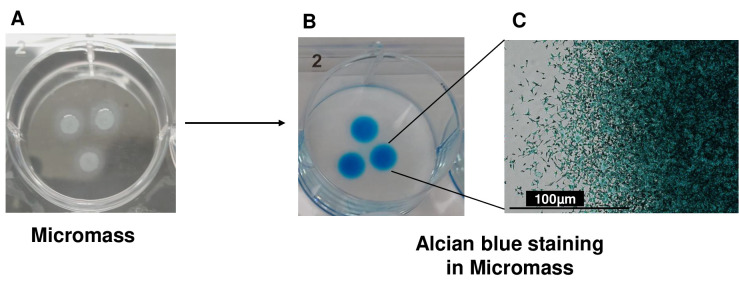
3D micromass culture for chondrogenic differentiation of induced pluripotent stem derived mesenchymal progenitor cells (iMSCs). (A) Chondrogenic differentiation of iMSCs using the micromass method (B) and Alcian blue staining for the assessment of proteoglycan deposition. Alcian blue staining showed accumulation of proteoglycans, indicating deposition of extracellular matrix in AC-iMSC micromass culture. (C) High magnification image demonstrating cellular compaction and condensation indicating the formation of chondrocytes. Scale bar, 100 μm.
  7. Allow the cells to attach for 2 h at 37 °C in a humidified 5% CO_2_ incubator. Following attachment, carefully apply 1.5 mL of MSC growth medium containing 10 μM of ROCK inhibitor Y-27632 to each well. Return the plate to the cell culture incubator and maintain overnight.
  8. After 24 h, aspirate the medium and add 2 mL of chondrogenic differentiation medium to each well. Continue incubation at 37 °C in a humidified 5% CO_2_ incubator.
  9. On day 2, add fresh chondrogenic differentiation medium containing BMP-2 or TGFβ3 to each well. Replace the growth factor-supplemented medium every other day.
**Pellet formation method**
  1. To initiate chondrogenic differentiation using the pellet formation method, harvest the iPSC-derived MSCs (iMSCs) using 0.25% trypsin-EDTA. Following centrifugation of the cells at 100× *g*, resuspend them in MSC growth medium.
  2. Perform viable cell counting via microscopy on a hemocytometer or other cell counting equipment using Trypan Blue solution in a 1:2 dilution of the cell suspension from step 1. Aliquot 2.5 × 10^5^ viable iMSCs in 0.5 mL of MSC growth medium into 15 mL conical tubes for pellet cultures. Centrifuge the cells at 100× *g* for 1 min and incubate them in the 15 mL conical tubes overnight at 37 °C and 5% CO_2 _with loosened caps to facilitate gas exchange.
  3. Within 24–48 h of pellet formation, verify that the cells have formed a spherical pellet at the bottom of each 15 mL tube (Figure 4). Figure 4 indicates the condensation of cells in the form of 3D-pellet culture at the bottom of 15 mL conical tube. If cells do not form a pellet under these conditions, coat a 15 mL conical tube with anti-adherence rinsing solution for 1 h, wash with PBS, and then add cells to allow pellet formation.
    **Figure 4.**
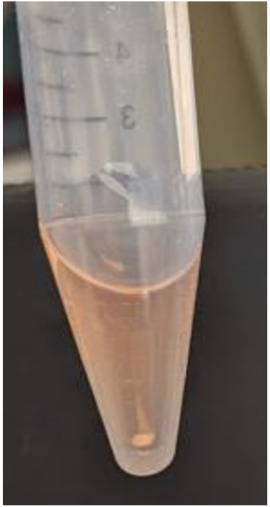
Chondrogenic differentiation of induced pluripotent stem cells (iMSCs) using pellet method
  4. Aspirate the medium and replace it with 0.5 mL of chondrogenic differentiation medium supplemented with either BMP-2 or TGFβ3.
  5. Subsequently, culture the pellets in chondrogenic medium with BMP-2/TGFβ3 for 7, 14, and 21 days, with careful medium exchange in each 15 mL conical tube using 0.5 mL of fresh chondrogenic medium containing BMP-2 or TGFβ3 every other day.
- **Histological assessment of chondrogenic matrix production**
  1. Harvest the cell pellets and wash them once in PBS. Subsequently, fix the pellets in formalin solution for a duration of 15 min.
  2. Perform two washes of the fixed pellets in PBS, followed by sequential dehydration steps in 1.5 mL microcentrifuge tubes using 50%, 70%, 95%, and 100% ethanol and then ethanol/xylene solution and 100% xylene. Each dehydration step should take 5 min (two repetitions, see Note 24). Embed the dehydrated pellets in low-melt paraffin.
  3. Use a microtome to section the paraffin-embedded pellets, creating sections of 5–7 μm thickness, and mount them onto Superfrost Plus slides.
  4. De-paraffinize and rehydrate the sections on slides by subjecting them to sequential washes in 100% xylene (two repetitions, each for 10 min), 100%, 95%, 70%, and 50% ethanol (two repetitions, each for 2 min), and finally, distilled water (for 5 min).
  5. To detect sulfated proteoglycan deposits indicative of functional chondrocytes, rinse the formalin-fixed sections of paraffin-embedded pellets in an acetic acid solution for 3 min. Subsequently, stain the sections with Alcian Blue solution at room temperature for 30 min. After a brief rinse in acetic acid solution and running tap water for 5–10 min, counterstain the cell nuclei with Nuclear Fast Red Kernechtrot solution at room temperature for 5 min, followed by a rinse in running tap water until clear (Figure 3B). Alcian blue staining as shown in Figure 3B shows accumulation of proteoglycans, which demonstrate the deposition of extracellular matrix in micromass culture of iMSCs. A high-magnification image demonstrates cellular compaction and condensation indicating the development of cartilage/chondrocytes (Figure 3C).
  6. For visualization of acidic proteoglycan in cartilage tissues, stain the hydrated formalin-fixed sections of paraffin-embedded pellets with Safranin O solution at room temperature for 5 min. Remove excess stain by rinsing the sections under running tap water for 5–10 min.
  7. Dehydrate the slides in 70%, 95%, and 100% ethanol, then 100% xylene (two repetitions, each for 2 min). Finally, mount coverslips using CytoSeal mounting medium to facilitate microscopic assessment of proteoglycan content, with Alcian Blue staining resulting in diffuse blue dye deposits, and Safranin O staining leading to diffuse orange-red deposits.
- **Quantitative PCR analyses of cartilage genes**
  For PCR analysis, RNA is extracted from cell pellets collected throughout the chondrogenic differentiation time course (refer to Note 13).
  1. Combine 2–3 pellets per experimental group with 1 mL of TRIzol and transfer the mixture to a sterile, RNase-, DNase-, and pyrogen-free 1.5 mL microcentrifuge tube.
  2. Pass the pellets in TRIzol reagent several times through a 26 1/2-gauge needle attached to a 1 mL syringe. Vortex the sample and incubate it at room temperature for 5 min.
  3. Add 200 μL of chloroform to the sample, vortex for 30 s, and incubate for 5 min.
  4. Centrifuge the samples at 12,000× *g* for 15 min at 4 °C to achieve phase separation. Place the tubes on ice.
  5. Transfer the upper aqueous RNA layers into a new 1.5 mL centrifuge tube.
  6. Add 500 μL of isopropanol to each sample, mix by inversion, and incubate at 4 °C for 30 min for RNA precipitation. At this stage, samples may be stored at -20 °C for extended periods.
  7. Centrifuge the samples at 12,000× *g* for 30 min at 4 °C. A small glassy oval/sphere will be strongly adhered to the side of the tube. Place the samples on ice. Wash the RNA pellets with 500 μL of 70% ethanol solution and spin at 12,000× *g* for 5 min at 4 °C. Aspirate the supernatant and repeat the washing step two times.
  8. Remove the ethanol and air-dry the RNA pellets. Dissolve the RNA pellets in an appropriate volume of ultrapure nuclease free water.
  9. Measure the RNA concentration for each sample using a NanoDrop^TM^ spectrophotometer.
  10. Treat the RNA samples with DNase I as per the manufacturer’s protocol and then perform reverse transcription of RNA to cDNA using the cDNA Reverse Transcription kit, following the manufacturer’s protocol.
  11. Conduct real-time quantitative PCR on the cDNA samples synthesized from total RNA using the SYBR Green Master Mix, along with gene-specific real-time PCR primers and a real-time PCR cycler according to the manufacturer’s protocol.
  12. Use the data obtained from quantitative RT-PCR analyses to calculate values represented as 2^-ΔΔCt^, wherein ΔΔCt refers to the difference in crossing threshold (Ct) values between the experimental and control samples, employing β-actin as an internal standard. Data presented in Figure 5 show high expression of mesenchymal marker genes such as RUNX1 and COL1A1 in AC-iMSCs culture.
    **Figure 5.**
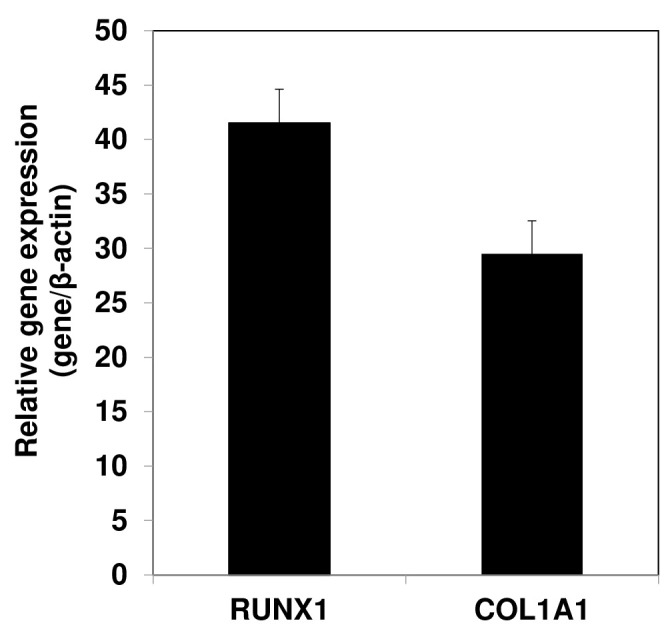
Gene expression analyses by qPCR showing expression of mesenchymal genes COL1A1 and RUNX1 in the AC-iMSCs relative to their parental induced pluripotent stem cells (iPSCs). β-Actin served as the housekeeping gene and internal control. Data represented as fold change relative to respective parental iPSCs.
  13. Present the data as mean ± S.E.M. of at least three independent samples. Perform statistical comparisons between untreated and growth factor–treated groups using a two-tailed Student’s *t*-test. Significance is assigned to P values < 0.05 (see Note 14).

## Data analysis

1. Flow cytometric analysis of MSCs surface marker should be analyzed counting the percentage of cells expressing the cell surface markers using FloJo software.
2. For the gene expression analysis, Ct value was used to calculate values represented as 2^-ΔΔCt^, wherein ΔΔCt refers to the difference in crossing threshold (Ct) values between the experimental and control samples.
3. Present the data as mean ± S.E.M. of at least three independent samples. Perform statistical comparisons between untreated and growth factor–treated groups using a two-tailed Student’s *t*-test. Significance is assigned to P values < 0.05.

## Validation of protocol

This protocol or parts of it has been used and validated in the following research article(s):

- Khan, N. M., Diaz-Hernandez, M. E., Chihab, S., Priyadarshani, P., Bhattaram, P., Mortensen, L. J., Guzzo, R. M. and Drissi, H. (2023). Differential chondrogenic differentiation between iPSC derived from healthy and OA cartilage is associated with changes in epigenetic regulation and metabolic transcriptomic signatures. *eLife*

## General notes and troubleshooting

**General notes**

1. Avoid keeping the mTeSR^TM^ Plus medium in a 37 °C water bath. This may affect the performance of the components present in mTeSR^TM^ medium; these components may also precipitate due to large temperature change from 4 °C to 37 °C. Instead, warm the mTeSR^TM^ Plus medium at room temperature (15–25 °C).
2. Transfer iPSCs into the Falcon tube using a drop-wise method while gently moving the Falcon tube back and forth to mix the iPSCs. This approach minimizes osmotic shock to the cells and ensures their viability.
3. To enhance the plating efficiency and viability of iPSCs after thawing, add Rock inhibitor Y-27632 (1,000× stock solution) to the iPSC medium 24 h prior to the procedure, without affecting their pluripotency.
4. Handle culture plates with care, gently moving them without swirling to prevent cell accumulation in the center of the plates.
5. After thawing, expect visible iPSC colonies within two days; after 4–5 days, the iPSCs should be ready for passaging.
6. When using mTeSR^TM^ Plus medium system, avoid using enzymes like collagenase IV or dispase for iPSCs dissociation. Instead, opt for ReLeSR, an enzyme-free reagent suitable for routine passaging, which eliminates the need for manual removal of differentiated regions.
7. Depending on the iPSC lines and colony quality, the treatment time of ReLeSR may vary, and for specific iPSC lines, the optimal dissociation time with ReLeSR could take up to 5 min.
8. After ReLeSR treatment, gently pipette the cell suspension multiple times to break up iPSC colonies into small cell clumps, being cautious not to create single-cell suspensions.
9. The split ratio for different iPSC lines can vary. Established iPSC cultures can be split with a ratio of 1:3 to 1:6, meaning cell clumps from one well can be plated into three to six wells.
10. Avoid disturbing the plate overnight to ensure maximum attachment of cells and prevent uneven distribution of cell clumps, which may lead to increased iPSC differentiation.
11. Some cell debris the day after passaging is normal. iPSCs can be passaged or frozen down with mTeSR^TM^ Plus freezing medium when they reach 80% confluence. Avoid over-confluence to prevent spontaneous differentiation.
12. For MSC differentiation, iPSCs should be at least 40% confluent two days after seeding. If there are insufficient cells for differentiation initiation, incubate them for one or two additional days, but avoid over-confluence before MSC differentiation to prevent random differentiation.
13. During the initial days of iPSC differentiation into MSCs, wash the differentiated cells with DPBS w/o Ca^2+^ and Mg^2+^ when renewing the medium due to a high number of dead cells. Subsequently, if the spent medium is clear and there are fewer dead cells, washing the cells becomes unnecessary.
14. Culture cells on gelatin-coated plates up to passage 2; as passaging continues, cells acquire a homogeneous, fibroblast-like morphology on gelatin-coated tissue culture plates. Beyond passage 2–3, use tissue culture plates without gelatin coating.
15. This methodology has been successfully employed in our lab to generate mesenchymal-like progenitors from various sources of human iPS cells. The multilineage differentiation potential of human iMSCs, including osteogenesis, adipogenesis, and chondrogenesis, has been established in vitro (Khan et al., 2023).
16. A cell density of 1 × 10^4^ cell/cm^2^ is recommended for the growth of MSCs derived from human iPSCs. At this stage, cells can be directly split into new T25 flasks at a ratio of 1:3–1:4.
17. When iPSC-MSCs (iMSCs) reach 80% confluence in a 10 cm culture dish, they can be frozen into 3–5 cryogenic vials.
18. Remember to include groups for unstained control and negative control (isotype-matched antibodies, such as IgG1-PE and IgG2b-FITC) during experimentation.
19. Ensure that MSCs are in a single-cell suspension to prevent clogging of the flow tubing and to avoid damage to the flow cell instrument.
20. For optimal results, it is recommended to analyze samples on the same day. If immediate analysis is not possible, store the samples in the dark at 4 °C for several days until analysis.
21. Use enzyme solutions (trypsin-EDTA, ReLeSR) and PBS at ambient temperature.
22. To promote optimal micromass attachment, it might be necessary to coat the plates with 0.1% gelatin. Ensure thorough drying of the plates before micromass formation to prevent cell spreading.
23. Prevent micromasses from drying during the 2 h incubation period by adding PBS to the reservoir between wells.
24. To facilitate visualization of the pellets during paraffin sectioning, a brief addition of Eosin or Alcian blue stain at 1:100 to the final 95% ethanol wash can provide color before complete dehydration in 100% ethanol and xylene.
25. During handling of the cryogenic vials from liquid N_2_ tank, always wear proper personal protective equipment (PPE) such as safety goggles and/or face shield and cryogen gloves; do not leave skin exposed. Do not wear metal jewelry or watches.
